# Postsynaptic localization of PSD-95 is regulated by all three pathways downstream of TrkB signaling

**DOI:** 10.3389/fnsyn.2014.00006

**Published:** 2014-03-31

**Authors:** Akira Yoshii, Martha Constantine-Paton

**Affiliations:** ^1^Department of Anatomy and Cell Biology, University of Illinois at ChicagoChicago, IL, USA; ^2^McGovern Institute for Brain Research, Massachusetts Institute of TechnologyCambridge, MA, USA; ^3^Constantine-Paton Laboratory, Department of Brain and Cognitive Science, McGovern Institute for Brain Research, Massachusetts Institute of TechnologyCambridge, MA USA; ^4^Department of Biology, McGovern Institute for Brain Research, Massachusetts Institute of TechnologyCambridge, MA, USA

**Keywords:** synapse formation, BDNF, TrkB, PSD-95, PKMϖ, protein kinase C, MAP kinase, PI-3 kinase

## Abstract

Brain-derived neurotrophic factor (BDNF) and its receptor TrkB regulate synaptic plasticity. TrkB triggers three downstream signaling pathways; Phosphatidylinositol 3-kinase (PI3K), Phospholipase Cγ (PLCγ) and Mitogen activated protein kinases/Extracellular signal-regulated kinases (MAPK/ERK). We previously showed two distinct mechanisms whereby BDNF-TrkB pathway controls trafficking of PSD-95, which is the major scaffold at excitatory synapses and is critical for synapse maturation. BDNF activates the PI3K-Akt pathway and regulates synaptic delivery of PSD-95 via vesicular transport (Yoshii and Constantine-Paton, 2007). BDNF-TrkB signaling also triggers PSD-95 palmitoylation and its transport to synapses through the phosphorylation of the palmitoylation enzyme ZDHHC8 by a protein kinase C (PKC; Yoshii etal., 2011). The second study used PKC inhibitors chelerythrine as well as a synthetic zeta inhibitory peptide (ZIP) which was originally designed to block the brain-specific PKC isoform protein kinase Mϖ (PKMϖ). However, recent studies raise concerns about specificity of ZIP. Here, we assessed the contribution of TrkB and its three downstream pathways to the synaptic distribution of endogenous PSD-95 in cultured neurons using chemical and genetic interventions. We confirmed that TrkB, PLC, and PI3K were critical for the postsynaptic distribution of PSD-95. Furthermore, suppression of MAPK/ERK also disrupted PSD-95 expression. Next, we examined the contribution of PKC. While both chelerythrine and ZIP suppressed the postsynaptic localization of PSD-95, RNA interference for PKMϖ did not have a significant effect. This result suggests that the ZIP peptide, widely used as the “specific” PKMϖ antagonist by many investigators may block a PKC variant other than PKMϖ such as PKCλ/ι. Our results indicate that TrkB regulates postsynaptic localization of PSD-95 through all three downstream pathways, but also recommend further work to identify other PKC variants that regulate palmitoylation and synaptic localization of PSD-95.

## INTRODUCTION

The Brain-derived neurotrophic factor (BDNF) and its receptor TrkB are critical for maturation of both excitatory (Gorski et al., 2003; Wirth et al., 2003; Chakravarthy et al., 2006; Tanaka et al., 2008; Kaneko et al., 2012) and inhibitory neurons (Hanover et al., 1999; Huang et al., 1999). In the visual cortex, BDNF increases 2 weeks after birth largely due to the visual activity after eye opening (Castren et al., 1992). BDNF-TrkB signaling regulates excitation–inhibition balance (Schinder et al., 2000) and facilitates activity-dependent formation of neuronal circuits (Cabelli et al., 1995, 1997; Heimel et al., 2010) as well as critical period closure of ocular dominance (Hanover et al., 1999; Huang et al., 1999).

In excitatory neurons, BDNF-TrkB signaling regulates dendritic growth (Xu et al., 2000), spine maturation, stabilization (Gorski et al., 2003; Wirth et al., 2003; Chakravarthy et al., 2006; Tanaka et al., 2008; Kaneko et al., 2012), and long-term potentiation (LTP; Kang and Schuman, 1995; Figurov et al., 1996; Patterson et al., 1996; Tanaka et al., 1997; Frerking et al., 1998; Gottschalk et al., 1998; Huber et al., 1998). BDNF-TrkB signaling also plays a critical role in the development of synapses by regulating the transport of the membrane associated guanylate kinase the post-synaptic density protein PSD-95. In the visual pathway of rodents upon eye-opening, PSD-95, which is the major scaffolding protein at mature glutamate synapses (Yoshii and Constantine-Paton, 2007; Yoshii et al., 2011), is transported to young synaptic contacts by BDNF/TrkB. SAP102, the dominant MAGUK in neonatal period, does not require an activation of BDNF-TrkB signaling for its postsynaptic localization (Yoshii and Constantine-Paton, 2007) and moves to early synapses in association with the NR2B subunit GluN2B (Washbourne et al., 2004). In our previous work, we reported two distinct mechanisms whereby the BDNF-TrkB pathway controls trafficking of PSD-95. BDNF activates the Phosphatidylinositol 3-kinase (PI3K)-Akt pathway and triggers synaptic delivery of PSD-95 via vesicular transport (Yoshii and Constantine-Paton, 2007). BDNF-TrkB signaling is also necessary for PSD-95’s initial association with membranes. It initiates PSD-95 palmitoylation through the phosphorylation of the palmitoylation enzyme ZDHHC8 by Phospholipase Cγ (PLC γ) and a protein kinase C (PKC; Yoshii et al., 2011). Here we verify our previous observations on the roles of the PI3K-Akt and PLC γ- PKC pathways in the transport of PSD-95 to synapses. We also extend these analyses of BDNF-TrkB signaling to the role of the third pathway downstream of TrkB activation: namely, the Mitogen activated protein kinase/Extracellular signal-regulated kinase (MAPK/ERK) pathway. Finally, in light of recent publications indicating that ZIP, the reagent we used to block the C kinase PKMϖ, is not specific for PKMϖ in LTP, (Lee et al., 2013; Volk et al., 2013) we test whether another PKC may also be involved in the palmitoylation of PSD-95.

## MATERIALS AND METHODS

### ANIMALS

TrkB^ F616A^ mice carrying a modification in the ATP binding site of this kinase (Chen et al., 2005) were kindly provided by Dr. David Ginty. Wild type (WT) C57BL6 mice were obtained from Charles River Laboratories. All manipulations were performed in accord with the guidelines of the MIT-IACUC.

### PRIMARY NEURON CULTURE, LIPOFECTION, IMMUNOCYTOCHEMISTRY

Occipital cortices of E15.5 mouse brains were dissected, digested with a solution containing papain and DNase for 25 min. Cells were dissociated using fire polished glass pipets and plated at the density of 0.5 × 10^6^ cells per cm^2^ after counting with a hemocytometer. Coverslips were coated with laminin and poly-D-lysine. Transfections of DNA constructs encoding either small interfering RNA (siRNA) against PKMϖ or its scrambled sequence (kindly provided by Dr. Richard Huganir, Johns Hopkins University) was performed at day *in vivo* (DIV) 8 using Lipofectamine 2000 (Invitrogen) according to the manufacturer’s protocol. On DIV15, cultured neurons were treated with the following reagents. 1NM-PP1 to block activation of TrkB or its control construct Bph-PP1 (kindly provided by Dr. Kevan Shokat, UCSF). U73122 (1 μM), Chelerythrine (2.5 μM), PD98059 (50 μM), and Wortmannin (100 nM) were used to block PLC, PKC MAPK, and PI3K, respectively, and were purchased from Sigma-Aldrich. The PKMϖ inhibitory pseudosubstrate ZIP (myr-SIYRRGARRWRKL-OH) and scrambled peptide (myr-RLYRKRIWRSAGROH; Pastalkova et al., 2006) were synthesized in the MIT Biopolymers Laboratory.

Twenty-four hours after each exposure, cultures were fixed with 4% paraformaldehyde (15 min). Neurons were permeabilzed with 0.3% TritonX-100 for 5 min and washed. Cultures were blocked with 9% BSA (1 h), and incubated with an antibody for PSD-95 (Neuromab #K28/43, 1:1000) at room temperature overnight. After washing with PBS (3x), cultures were incubated with secondary antibody Alexa Fluor 488 (Molecular Probes) for 2 h.

Images were captured using a 60x objective on a Nikon PCM 2000 confocal microscope and a 6–8 μm, Z-series, of optical sections were taken at intervals of 0.5 μm. 12/4 μm areas containing isolated typical secondary dendritic branches from pyramidal neurons were selected. For each area, a threshold was set to optimally represent PSD-95 puncta and exclude diffuse label in dendritic shafts (See **Figure 2A** The same threshold was applied to all neurons in the set of cultures in each experiment. Immunocytochemical experiments were repeated twice for each treatment condition. Eight cells from eight culture dishes were imaged. Using ImageJ, total pixel intensity was calculated as the sum of each pixel number multiplied by its intensity as measured above threshold for each cell. This value was then averaged across all dendritic segments sampled in the experiment and presented as total pixel intensity of PSD-95 puncta. Averaged cell body intensity was also measured in ImageJ by manually selecting soma (yellow circles in **Figure 2A**) and applying the Measure function in ImageJ. Subsequently, total pixel intensity of PSD-95 puncta was divided by the averaged cell body intensity for normalization.

### TUNEL ASSAY

FragEL^ TM^ DNA Fragmentaion Detection Kit (Calbiochem) was used for the TUNEL assay. The procedures were performed according to the manufacturer’s instructions. Cell numbers in 250 μm × 250 μm were counted and the apoptosis incidence was calculated by dividing the TUNEL (+) cell number with total cell number.

### STATISTICS

A Student’s *t*-test was used for comparison of two groups. One-way ANOVA with *post hoc* Tukey tests were used for comparisons of more than two groups. *p* < 0.05 were considered significant and indicated as ^*^ in graphs. *p* < 0.01 and *p* < 0.001 are indicated as ^**^ and ^***^. Numeric data are presented as average ±SD in the “Results” section. Error bars in graphs represent SEM.

## RESULTS

We previously studied trafficking of PSD-95 in dendrites of cultured occipital cortical neurons using Fluorescent recovery after photobleaching (FRAP) and showed that bath application of BDNF as well as a BDNF-coated beads facilitated transport of GFP-tagged PSD-95 in dendrites (Yoshii and Constantine-Paton, 2007). Furthermore, BDNF application resulted in an increase of the intensity of PSD-95 immunolabeled puncta.

**FIGURE 1:**
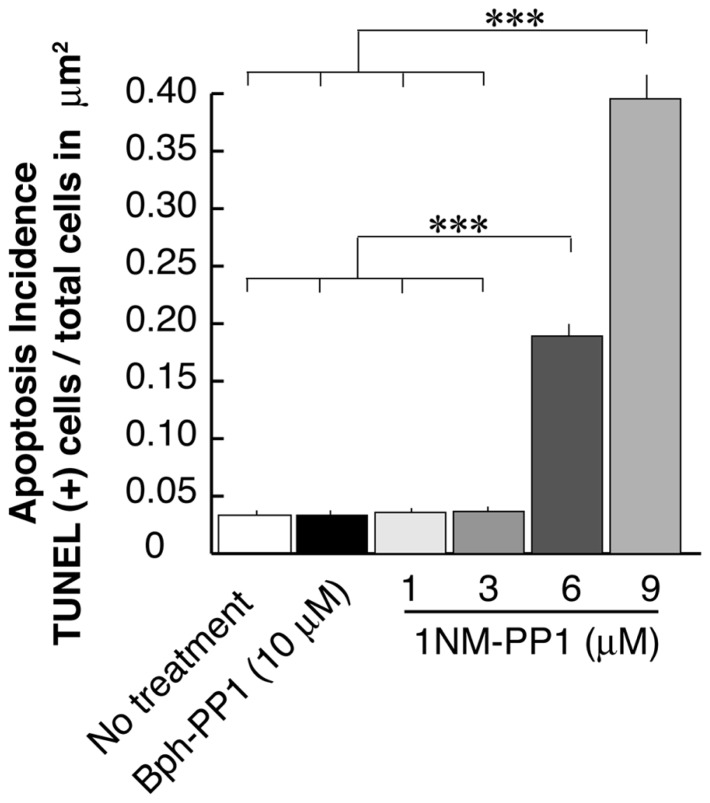
**Apoptosis rate of neurons that undergo TrkB signaling inhibition using the chemical genetic approach.** Ratio’s of Tunel signal positive cells to total cell number (~3,500–6,600 cells examined in 250 μm × 250 μm from eight cultures for each condition) are presented. Error bars represent SEM.

**FIGURE 2:**
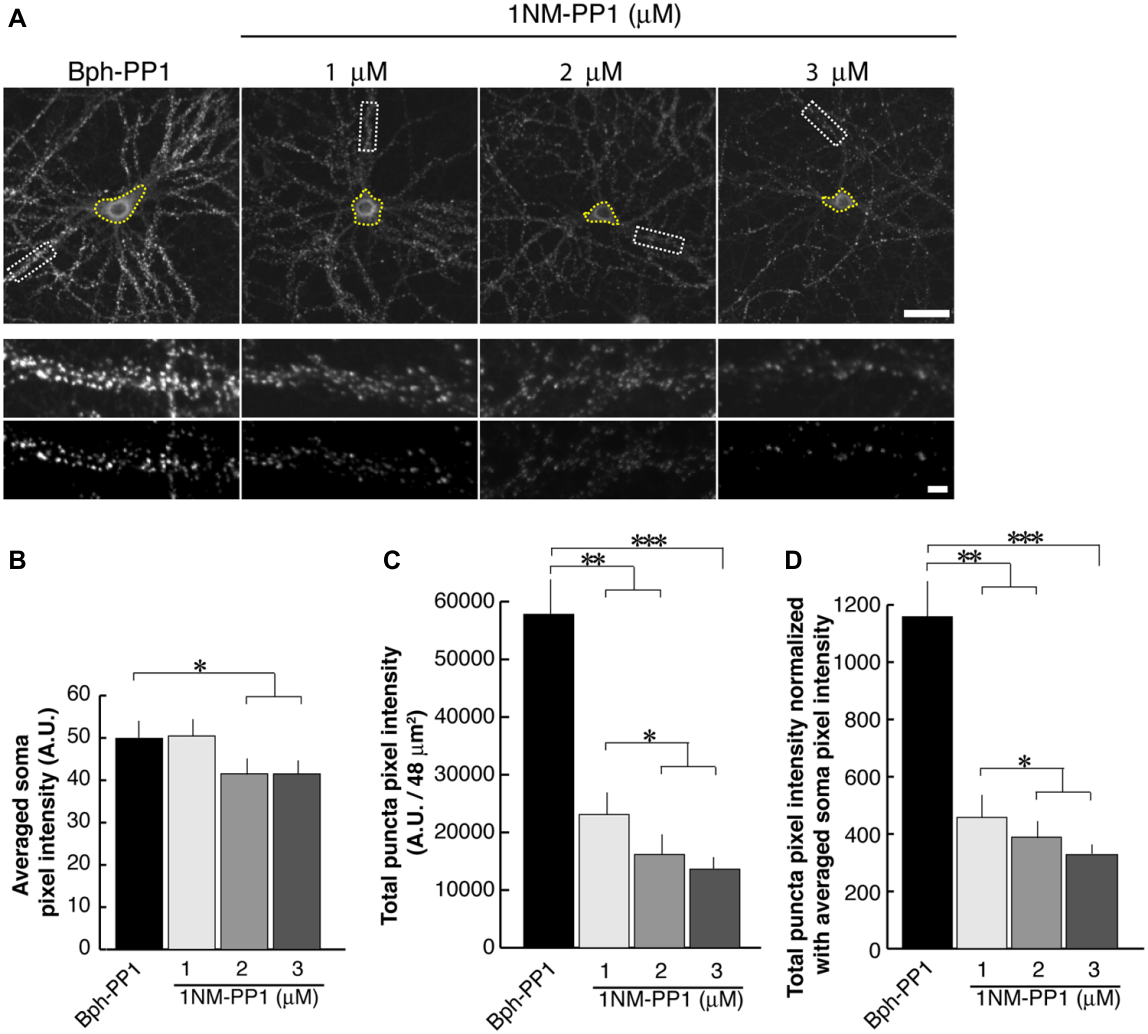
**1NM-PP1 suppresses punctate distribution of PSD-95 dose-dependently in TrkB^**F616A**^ neurons.** **(A)** The top row shows representative neurons immunostainted with anti-PSD-95. Typical secondary dendritic branches analyzed are shown in 48 μm^2^ rectangles in the middle row. These branches are processed after the thresholding shown in the bottom row. The scales in the top and bottom rows show 10 and 1 μm, respectively. **(B)** Graph showing averaged PSD-95 intensities in somata. **(C)** Graph showing quantification of PSD-95 puncta total pixel intensities. Note that all three concentrations of 1NM-PP1 result in reduced total PSD-95 puncta intensity. **(D)** In this graph, PSD-95 puncta intensities are normalized to somal PSD-95 intensities. In each condition, 16 branches from eight cells (chosen from the two different dissociations) were analyzed. Error bars represent SEM.

In the current experiments we first examined the postsynaptic distribution of PSD-95 in dissociated cultured neurons prepared from E15.5 cortices of TrkB^ F616A^ mice. This mouse strain has a single amino acid mutation in the intracellular domain of the TrkB receptor allowing the signaling activities of the receptor to be selectively blocked by the synthetic compound 1NM-PP (Chen et al., 2005). At DIV 15, we treated neurons with either 1NM-PP1 or the “control” non-blocking molecule Bph-PP1for 24 h. Using the TUNEL assay, we examined the incidence of apoptotic cells at various concentrations of 1NM-PP1 (**Figure 1**). We found that the cell death incidence was not significantly different at 1 (0.036 ± 0.020; total of 123 apoptotic cells out of 3430 cells in 33 images) and 3 μM (0.036 ± 0.021; total of 244 apoptotic cells out of 6652 cells in 33 images) as compared with no treatment (0.033 ± 0.025; total of 132 apoptotic cells out of 3961cells in 33 images) (ANOVA relative the no treatment group or the control Bph-PP1 group *p *= 0.96 and 0.79, respectively). We determined 9 μM of 1NM-PP1 is toxic because 40% of cells are TUNEL^+^. Consequently, PSD-95 puncta intensity was accessed using 1NM-PP1at 3 μM or less. First, we measured the averaged pixel intensity of the cell bodies (encircled areas with yellow dashed lines in **Figure 2A**) and found that PSD-95 expression level was slightly but significantly decreased in cultures treated with 2 (41.5 ± 7.6; *N* = 8) or 3 μM (41.5 ± 8.7; *N* = 8) of 1NM-PP1 as compared with 1 μM (50.4 ± 10.1; *N* = 8) or Bph-PP1 control (49.8 ± 10.5; *N* = 8). Therefore, we normalized the total pixel intensity of PSD-95 puncta by the intensity of soma. First we selected the defined area (4 μm × 12 μm squares in **Figure 2A**) of secondary dendritic branches and acquired total pixel intensities of PSD-95 puncta (See Materials and Methods; Yoshii and Constantine-Paton, 2007; Yoshii et al., 2011). These crude PSD-95 total pixel intensities were significantly reduced in neurons treated with 1NM-PP1 (1 μM; 57,800 ± 23,200; *N* = 16) as compared to neurons without treatment (23,100 ± 11,600; *N* = 16; **Figure 2C**; ANOVA between 1 μM and the no treatment groups; *p *= 0.0043). Next, each intensity value was divided by averaged somal PSD-95 intensity of the same neuron. Normalized PSD-95 total pixel intensities remained significantly reduced in neurons treated with 1NM-PP1 as compared to control neurons treated with Bph-PP1 (**Figure 2D**; ANOVA between 1 μM and the control groups; *p *= 0.0089). Higher concentration of 1NM-PP1 caused more suppression of PSD-95 puncta (ANOVA between 1 and 3 μM groups; *p *= 0.043; **Figure 2D**).

**FIGURE 3:**
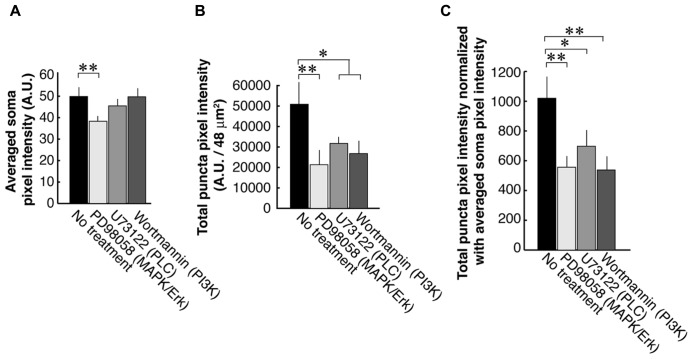
**PSD-95 puncta intensities in neurons that are treated with inhibitors of signaling molecules downstream of TrkB.** Neurons are treated with blockers of the PLC (U73122, 1 μM), MAPK (PD98059, 50 μM), or PI3K (Wortmannin, 100 nM). Quantification was performed in the same manner as data presented in **Figure 2**. The graphs show averaged PSD-95 intensities in somata **(A)**, total pixel intensities of PSD-95 puncta **(B)**, and puncta intensities normalized to somal intensities **(C)**. In each condition, 16 branches from eight cells were analyzed. Error bars represent SEM.

To examine each of the three TrkB downstream signaling pathways, we used antagonists against PLC, PI3K, and MAPK/ERK (**Figure 3**). First, we examined the PSD-95 immunolabel intensities of the somata and found that the blocker of the MAPK/ERK (38.3 ± 6.2; *N* = 8) but not two other signaling pathways (PI3K; 49.7 ± 7.4; *N* = 8 and PLC; 45.5 ± 4.7; *N* = 8) resulted in a decrease of somal PSD-95 expression (Control; 49.9 ± 10.5; *N* = 8; **Figure 3A**). Next we examined PSD-95 total puncta intensities in dendrites (**Figure 3B**). As expected from our previous works, blockade of PLC with U73122 (31,700 ± 10,200; *N* = 16) or PI3K with Wortmannin (23,100 ± 11,600; *N* = 16) caused suppression of PSD-95 puncta intensities as compared to the no treatment controls (50,900 ± 35,100; *N* = 16; ANOVA; *p *= 0.042 and 0.025, respectively). Furthermore inhibition of the MAPK/ERK pathway with PD98059 (21,300 ± 19,500; *N* = 16) resulted in a decrease of PSD-95 puncta intensities (ANOVA to the no treatment group; *p *= 0.0017). Normalization to averaged cell body intensity also confirmed that all three downstream pathways were involved in the normal postsynaptic delivery and/or expression of PSD-95 (**Figure 3C**).

**FIGURE 4:**
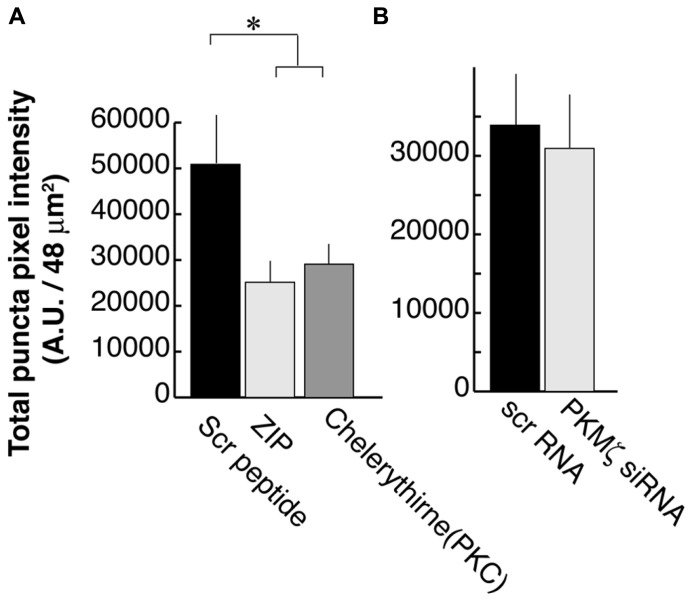
**Zeta inhibitory peptide (ZIP) but not PKMζ knockdown causes suppression in PSD-95 puncta intensity.** **(A)** Neurons are treated with ZIP (1 μM), PKC (Chelerythrine, 2.5 μM), or the scrambled ZIP peptide (1 μM). **(B)** Neurons are transfected with DNA constructs encoding siRNA for PKMϖ or the scrambled sequence. In both experiments, 16 branches from eight cells were analyzed. Error bars represent SEM.

We previously showed that activation of PLC by BDNF-TrkB signaling is necessary for PSD-95 palmitoylation and its transport to synapses. This mechanism depends on the phosphorylation of the palmitoylation enzyme ZDHHC8 by a PKC (Yoshii et al., 2011), which is activated by PLC. In that study, we used the PKC inhibitor Chelerythrine as well as a synthetic PKMϖ inhibitory peptide ZIP which was originally thought to specifically block this brain-specific PKC isoform (Ling et al., 2002). We confirmed that ZIP treatments resulted in a reduction of PSD-95 puncta intensity (25,100 ± 13,900; *N* = 16; **Figure 4A**; ANOVA to control; *p *= 0.02) as was also reported in the hippocampus (Shao et al., 2012), subsequent to our original finding in the visual cortex. Chelerythrine treatment also showed a similar result as ZIP treatment (28,000 ± 13,000; *N* = 16; ANOVA to control; *p *= 0.035).

However, in contrast to earlier experiments suggesting that ZIP was a specific PKMϖ antagonist (Ling et al., 2002), recent studies using PKMϖ knockout mice have not shown the expected LTP defect even though they did confirm that ZIP suppressed the LTP response (Lee et al., 2013; Volk et al., 2013). These reports raised the concern about the specificity of ZIP for PKMϖ. Consequently, we tested PKMϖ directly by suppressing its expression using RNAi. We transfected DNA constructs encoding siRNA against PKMϖ or the scrambled sequence at DIV8 and examined it at DIV16. RNAi for PKMϖ did not have a significant effect on postsynaptic localization of PSD-95 (30,600 ± 21,200; *N* = 16) as compared to neurons transfected with the scrambled sequence construct (33,600 ± 20,300; *N* = 16; Student’s *t*-test; *p *= 0.73; **Figure 4B**).

## DISCUSSION

### SYNAPTIC EXPRESSION OF PSD-95 DEPENDS ON PKC BUT NOT PKMζ

In our previous *in vitro *study, we applied BDNF to neurons expressing PSD-95 tagged with GFP and saw an increase in puncta intensity in 60 min (Yoshii and Constantine-Paton, 2007). We also used FRAP and pharmacological treatments and showed that the BDNF effect on vesicular transport of PSD-95 requires the PI3K/Akt pathway (Yoshii and Constantine-Paton, 2007). In the following *in vivo *study, we used TrkB^ F616A^ mice and showed that our synchronized eye-opening regime, which triggers PSD-95 redistribution to synapses in rats (Yoshii et al., 2003), also activates TrkB in visual cortex and regulates the synaptic localization of PSD-95. Furthermore, the TrkB activation is necessary for palmitoylation of PSD-95 in somata through PLC, PKC, and the palmitoylation enzyme ZDHHC8 (Yoshii et al., 2011). In the current study on cultured visual cortical neurons using the same pharmacological intervention, we confirmed that postsynaptic distribution of PSD-95 depends on BDNF-TrkB signaling, and that PI3K and PLC are necessary for increased PSD-95 at synaptic puncta. However, even though ZIP suppressed PSD-95 puncta intensity, knockdown of PKMϖ using RNAi has no effect on postsynaptic expression of PSD-95. This result is consistent with recent reports showing PKMϖ knockout mice do not show an abnormal LTP response (Lee et al., 2013; Volk et al., 2013). One possibility for these LTP results, suggested by Lisman (2012), is that the concentration of ZIP is too high and causes non-specific inhibition (Lisman, 2012). Alternatively, ZIP may not be a specific PKMϖ inhibitor rather it may also interact with other PKC variants such as another atypical PKC (PKCλ/ι; Lee et al., 2013; Volk et al., 2013). These results necessitated revising our previous interpretation (Yoshii et al., 2011). The current results suggest that a PKC variant other than PKMϖ should be present in the developing visual cortex and is likely to phosphorylate the palmitoylation enzyme ZDHHC8.

### MAPK/ERK IS INVOLVED IN SYNAPTIC EXPRESSION OF PSD-95

The current work also indicates that the MAPK/ERK pathway plays a role in the synaptic expression of PSD-95 as its inhibitor PD98059 significantly suppresses PSD-95 intensities of the soma as well as dendrite. This is consistent with the previous report showing that BDNF-induced increase in dendritic spine density is mediated by MAPK/ERK1/2 (Alonso et al., 2004). MAPK/ERK regulates protein-synthesis dependent plasticity by increasing phosphorylation of eukaryotic initiation factor 4E (eIF4E), the 4E-binding protein 1 (4E-BP1) and ribosomal protein S6 (Kelleher et al., 2004; Klann and Dever, 2004), therefore this pathway may directly initiate translation of PSD-95 gene either in the cell bodies of young neurons or in response to local activity in dendritic spines.

Brain-derived neurotrophic factor also activates the elongation of translation, which is mediated by PI3K and MAPK/ERK (Inamura et al., 2005). These two kinases turn off the eukaryotic elongation factor 2 kinase (eEF2K), in two parallel mechanisms. In turn, the suppression of eEF2K results in an increase of dephosphorylated and active eukaryotic elongation factor 2 (eEF2; Wang et al., 2001). This laboratory previously showed that NMDAR activation and visual activity rapidly induces phosphorylation of eEF2 at the synapse (Scheetz et al., 1997). This suppresses synthesis of most proteins but facilitates CaMKII synthesis. In rat dentate gyrus, BDNF application *in vivo* has been shown to induce LTP via ERK pathway, increase phosphorylation of eEF2 at non-synaptic sites and enhance expression of CaMKII-α and Arc (Kanhema et al., 2006). Furthermore, Worley and his colleagues obtained similar results for eEF2 phosphorylation via activation of mGluR5 in the hippocampus where the general shutdown of protein synthesis at the synapse significantly increased Arc/Arg3 translation (Park et al., 2008).

Another potential mechanism is that MAPK/ERK may be involved is mRNA transport. PSD-95 transcripts have been shown to exist in dendrites and interact with the fragile X mental retardation protein (FMRP). This enhances stability of the PSD-95 transcript and represses its translation during mRNA transport (Zalfa et al., 2007). A recent study shows that FMRP forms a complex with CYFIP1, a newly identified 4E-binding protein, and represses translation during mRNA transport (Napoli et al., 2008). BDNF can release this translational repression (Napoli et al., 2008). Whether MAPK/ERK meditates BDNF-dependent protein synthesis via translation and/or mRNA transport awaits future studies.

Brain-derived neurotrophic factor application also regulates transcription via MAPK/ERK which phosphorylates the cAMP-response element binding transcription factor (CREB) at serine133 residue (Bonni et al., 1995, 1999; Finkbeiner et al., 1997; Shaywitz and Greenberg, 1999; Pizzorusso et al., 2000; Ying et al., 2002). Interestingly, CREB can activate the *Bdnf* gene through promoter IV (Hong et al., 2008) and amplify BDNF-dependent synapse maturation. Therefore, we predict that MAPK/ERK regulates either or both transcription and translation of PSD-95. However, it remains to be studied whether CREB directly activates transcription of PSD-95 itself or up-regulates BDNF, which further facilitates posttranscriptional regulation of PSD-95, i.e., translation, palmitoylation, or vesicular transport.

## CONCLUSION

It is now clear that the increases in PSD-95 at synapses are mediated by all three signaling pathways downstream of TrkB. They are involved in various processes regulating protein expression. MAPK/ERK could regulate transcription through CREB and other transcription factors. MAPK/ERK and PI3K-Akt pathway play major roles in translation (Kelleher et al., 2004; Klann and Dever, 2004). The PI3K pathway also facilitates vesicular transport of PSD-95 from ER to Golgi apparatus, then along microtubule. PSD-95 is synthesized in the cytoplasm and requires palmitoylation to become attached to membranes and to get delivered to postsynaptic membranes. This post-translational modification is regulated by BDNF-TrkB signaling via PLC–PKC. These same mechanisms are likely to regulate BDNF-dependent long-term plasticity. Furthermore, PSD-95 itself and its interaction with TrkB signaling have been implicated in various brain diseases, especially neurodevelopmental disorders such as autism spectrum disorders (Tsai et al., 2012), Angelman syndrome (Cao et al., 2013), and schizophrenia (Mukai et al., 2008).

## Conflict of Interest Statement

The authors declare that the research was conducted in the absence of any commercial or financial relationships that could be construed as a potential conflict of interest.
